# Professionals’ Practices to Support Chinese-American Dementia Caregivers’ Decision-Making on Feeding

**DOI:** 10.1093/geroni/igaf122.1128

**Published:** 2025-12-31

**Authors:** Xiaoyi Zeng, Peiyuan Zhang, Jing Wang, Dena Schulman-Green, Bei Wu, Laura Hanson, Yaolin Pei

**Affiliations:** The University of Texas at Austin, Austin, Texas, United States; University of Maryland, Baltimore County, Baltimore, Maryland, United States; University of New Hampshire, Durham, New Hampshire, United States; New York University, New York, New York, United States; NYU Shanghai, Shanghai, China (People’s Republic); The University of North Carolina at Chapel Hill School of Medicine, Chapel Hill, North Carolina, United States; The University of Texas at Austin, Austin, Texas, United States

## Abstract

Health and social care providers play a crucial role in shared decision-making with caregivers, families, and older people with dementia regarding care options. However, less is known how these providers support the families of individuals with advanced dementia regarding decision-making on feeding options in Chinese American community. This qualitative study explored professionals’ practices in supporting Chinese American dementia caregivers’ decision-making on feeding options. We conducted individual, semi-structured interviews with thirteen professionals, with 69.2% having over ten years of professional experiences serving Chinese American older people with advanced dementia and their caregivers. Participants represented diverse disciplines and practice settings. Two bilingual researchers independently coded the transcripts and then jointly resolved discrepancies through iterative discussions. Thematic analysis identified four key professional practices: (1) assessing the patient’s condition and family dynamics; (2) providing emotional support to family caregivers while advocating for patients’ treatment preferences and goals of care; (3) offering continual and repeated education (e.g., on feeding options, eating management, disease progression, and end-of-life care); and (4) facilitating family communication and interdisciplinary collaboration to support informed decision-making. Decision-making was described as a dynamic, ongoing process shaped by sociocultural factors (e.g., the cultural importance of food) and family dynamics. Professionals also noted key challenges, such as time constraints and lack of advance directives. Findings highlighted the importance of provider-caregiver education, sociocultural considerations, and family-centered decision-making. They also underscored the need for dementia and end-of-life education, early advance care planning, and culturally tailored resources for Chinese American older people with dementia, their caregivers, and families.

